# Well-being is associated with cortical thickness network topology of human brain

**DOI:** 10.1186/s12993-023-00219-6

**Published:** 2023-09-25

**Authors:** Yubin Li, Chunlin Li, Lili Jiang

**Affiliations:** 1 CAS Key Laboratory of Behavioral Science, Institute of Psychology, Chinese Academy of Sciences, No. 16 Lincui Road, Chaoyang District, Beijing, 100101 China; 2 Department of Psychology, University of Chinese Academy of Sciences, Shijingshan, Beijing, China

**Keywords:** MRI, Well-being, Cortical thickness, Network efficiency, Network centrality

## Abstract

**Background:**

Living a happy and meaningful life is an eternal topic in positive psychology, which is crucial for individuals’ physical and mental health as well as social functioning. Well-being can be subdivided into pleasure attainment related hedonic well-being or emotional well-being, and self-actualization related eudaimonic well-being or psychological well-being plus social well-being. Previous studies have mostly focused on human brain morphological and functional mechanisms underlying different dimensions of well-being, but no study explored brain network mechanisms of well-being, especially in terms of topological properties of human brain morphological similarity network.

**Methods:**

Therefore, in the study, we collected 65 datasets including magnetic resonance imaging (MRI) and well-being data, and constructed human brain morphological network based on morphological distribution similarity of cortical thickness to explore the correlations between topological properties including network efficiency and centrality and different dimensions of well-being.

**Results:**

We found emotional well-being was negatively correlated with betweenness centrality in the visual network but positively correlated with eigenvector centrality in the precentral sulcus, while the total score of well-being was positively correlated with local efficiency in the posterior cingulate cortex of cortical thickness network.

**Conclusions:**

Our findings demonstrated that different dimensions of well-being corresponded to different cortical hierarchies: hedonic well-being was involved in more preliminary cognitive processing stages including perceptual and attentional information processing, while hedonic and eudaimonic well-being might share common morphological similarity network mechanisms in the subsequent advanced cognitive processing stages.

## Background

Living a happy and meaningful life is a major topic in positive psychology, which have a great impact on individuals’ physical and mental health and help people flourish in their lives, in their communities, and in the world [1–3]. Researchers have proved that healthy people with higher levels of well-being tend to have better emotional states, better interpersonal relationships, and stronger senses of belonging to a group [2, 4, 5], thus they were less likely to suffer from mental illnesses [6]. Compared with other models of well-being mostly focusing on emotional (or subjective) aspect of well-being, Keyes [7, 8] developed the mental health continuum model composed of three well-being components: emotional (subjective) well-being, psychological well-being, and social well-being. Specifically, emotional well-being reflect the hedonic aspect of well-being that encompassed pleasure attainment, positive affective states, and high levels of life satisfaction [9]. Psychological well-being and social well-being together are considered as eudaimonic well-being, which refer to the actualization of individuals’ potential or true value and evaluation of one’s circumstance and functioning in society [10, 11]. Previous studies have shown that these three dimensions of well-being were moderately correlated with each other, and they were interrelated but distinct constructs [12–14].

Recently, neuroimaging studies have used different experimental approaches to enrich our understanding of both anatomical and functional substrates of different dimensions of well-being and showed a variety of association results [15]. For instance, a result from an electroencephalography study showed that the greater left than right superior frontal activation was associated with the higher levels of both hedonic and eudaimonic well-being [16]. MRI studies revealed many correlations between different dimensions of well-being and human brain structural metrics [e.g., the regional gray matter volume (rGMV) or regional gray matter density (rGMD)]. In more detail, social well-being was correlated with both rGMV in the left dorsolateral prefrontal cortex [17] and rGMD in the left orbitofrontal cortex [18], which were both involved in emotional regulation [19–21] and social-cognitive processes [22, 23]. Besides, several other studies reported the associations between emotional well-being and rGMV in the precuneus [24, 25], the rostral anterior cingulate [25, 26] and the left dorsolateral prefrontal cortex [26, 27]; as well as the correlation between psychological well-being and rGMV in the insula [27, 28]. Meanwhile, several resting-state fMRI studies also reported the links between (1) emotional well-being and human brain functional measurements [e.g., regional homogeneity (ReHo) and amplitude of low-frequency fluctuations (ALFF)] in the prefrontal cortex [28–30], subjective well-being and the fractional ALFF in the right precentral gyrus [31], and emotional well-being and resting state functional measurements in the limbic regions including the posterior cingulate cortex [32], the thalamus, the hippocampus, and the amygdala [33]; and between (2) social well-being and ALFF in the temporal gyrus, the limbic regions including the anterior cingulate cortex, the insula and the thalamus [34]. As well as (3) functional connectivity within the limbic network such as the bilateral anterior insula [35], and within the default mode network which was responsible for the internal thoughts concerning selfness as well as memory construction [36–39] were correlated with well-being [32, 33, 40–44]. Taken together, previous studies mostly concentrated on emotional and social well-being but not examined all the three dimensions of well-being, and there were both distinct and common neural mechanisms in emotional and social well-being [15].

Complex network analysis can be used to characterize human brain connectivity within the whole brain network and enhance our comprehension of human brain network architecture [45, 46]. In this study we used our macro-scale morphological similarity network based on the distributions of cortical thickness [47] to explore the neural mechanisms of the three different dimensions of well-being. Network topology represents the full connection details of a network and can elucidate the complex connectome of the brain network [45, 48, 49]. Recently, researchers have proposed a large number of meaningful local and remote connectivity measurements to quantify the topological properties of complex brain networks based on graph theory [45, 47, 48], of which network centrality and efficiency are two commonly used topological measurements. On one hand, network centrality assesses the functional importance of brain regions and provides us convincing information on how brain regions play crucial roles in promoting functional integration or segregation within the whole brain network [48–50]. On the other hand, network efficiency, as a measure of functional integration and segregation, assesses the ability of information transfer within the brain network [51, 52]. In more detail, global efficiency corresponds to long-distance information interaction; nodal efficiency reflects the ability of information transfer in the given region (node) over the whole network and local efficiency reflects the specialization of a single node and functional segregation within the neighbors of a given node [51, 53]. Researchers have applied these topological measurements to investigate brain network topological mechanisms of various behaviors and diseases [47, 49, 54].

In the study, we recruited 67 healthy participants (aged 18–64), who finished structural MRI scanning, followed by the assessments of well-being including emotional, psychological, and social well-being, to explore human brain morphological network topological mechanisms of different dimensions of well-being. We firstly constructed human brain morphological similarity network of cortical thickness for each participant, and then calculated topological properties including network centralities and efficiencies. Human brain morphological similarity network characterized individual regional distribution similarity of morphology. We attempted to answer the following questions: whether there were associations between well-being and human cortical thickness similarity network topology? Did different dimensions of well-being correspond to topological characteristics of different brain regions encoded in cortical thickness similarity network?

## Results

**Fig. 1:**
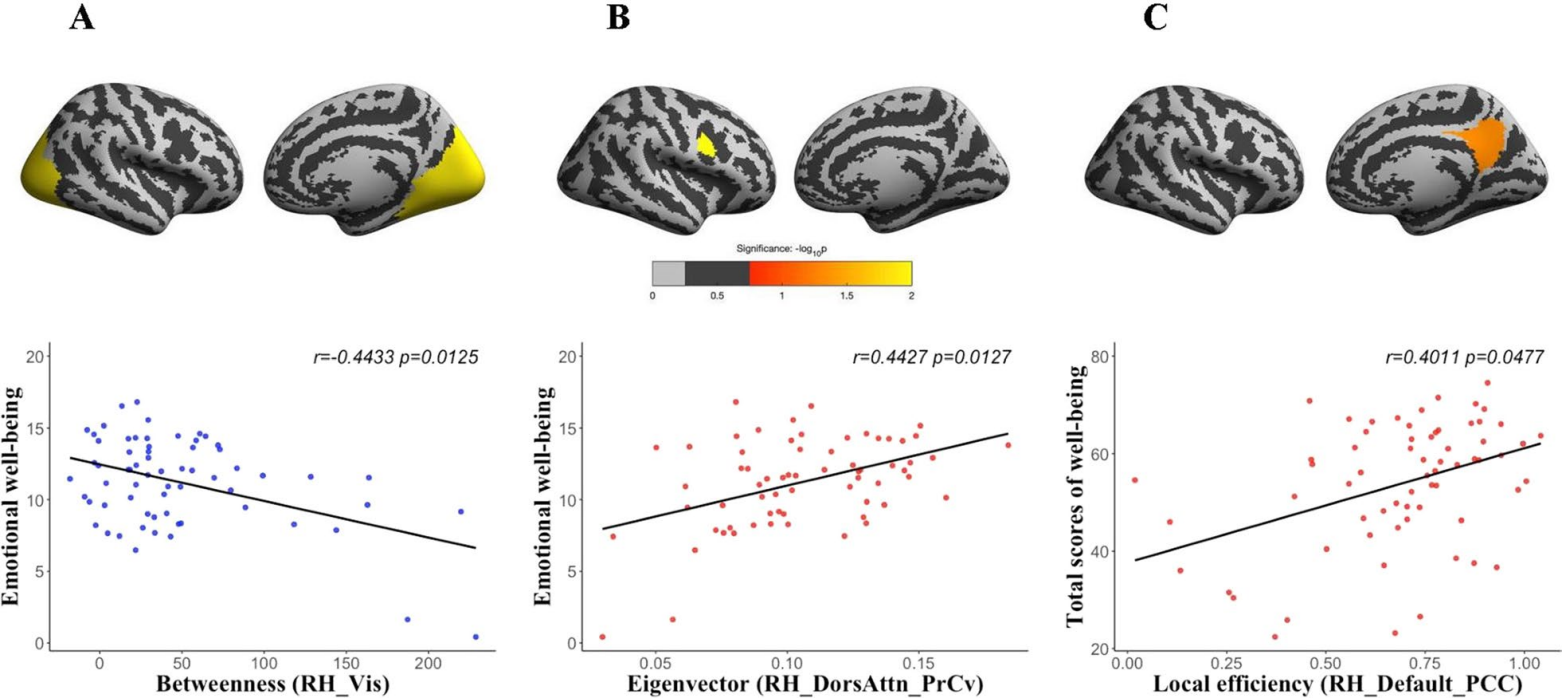
Brain regions that were significantly correlated with well-being. Betweenness centrality in the RH_Vis of cortical thickness network was negatively correlated with emotional well-being (**A**: r =  − 0.4433, corrected *p*-value = 0.0125). Eigenvector centrality in the RH_DorsAttn_PrCv of cortical thickness network was positively correlated with emotional well-being (**B**: r = 0.4427, corrected *p*-value = 0.0127). Local efficiency in the RH_Default_PCC of cortical thickness network was positively correlated with total scores of well-being (**C**: r = 0.4011, corrected *p*-value = 0.0477)

Table 1 illustrated detailed information about MHC-SF (the Mental Health Continuum, the Short Form) for the entire group including their average, standard deviation, maximum and minimum. There was no significant correlation between well-being and demographic variables such as age and education. Betweenness centrality reflects the important and bridging roles that connect disparate parts of the network, and we found emotional well-being was negatively correlated with the betweenness centrality in the RH_Vis (the visual network of the right hemisphere) of cortical thickness network (A: r = − 0.4433, corrected *p*-value = 0.0125, shown in Fig. 1a). Eigenvector centrality indicates a central and important role of the node within the network, and we found emotional well-being was positively correlated with the eigenvector centrality in the RH_DorsAttn_PrCv (the inferior part of the precentral sulcus) of cortical thickness network (B: r = 0.4427, corrected *p*-value = 0.0127, shown in Fig. 1b). Local efficiency reflects the ability of information transfer within the given brain region, and we found that total score of well-being was positively correlated with local efficiency in the RH_Default_PCC (the precuneus and the dorsal posterior cingulate cortex) of cortical thickness network (C: r = 0.4011, corrected *p*-value = 0.0477, shown in Fig. 1c), in which the effect sizes of emotional well-being, psychological well-being and social well-being were respectively 0.248, 0.341, and 0.340.

**Table 1:** Participant information: descriptive statistics and inter-variable correlations

Variables	Mean		Range	Age	Years of education	Total score of well-being	Emotional well-being	Psychological well-being
Age	37.29	13.11	18.59–64.3	1				
Years of education	15.37	3.19	8–22	− 0.401**	1			
Total score of well-being	53.88	13.17	19–70	0.101	− 0.126	1		
Emotional well-being	11.22	3.23	0–15	0.094	− 0.112	0.839**	1	
Psychological well-being	23.49	5.97	6–30	0.096	− 0.147	0.937**	0.700**	1
Social well-being	19.17	5.28	4–25	0.085	− 0.08	0.922**	0.688**	0.778**

*N* = 65; *p < 0.05; **p < 0.01

Our results showed that there were associations between well-being and human cortical thickness similarity network topology, and also different dimensions of well-being exhibited both common and different brain mechanisms encoded in cortical thickness similarity network.

## Discussion and conclusions

In the study, we applied a novel method to construct human brain morphological similarity network to investigate topological mechanisms of well-being in healthy participants. Compared with temporal synchronism encoded in human brain functional network, our morphological similarity network characterized individual regional distribution similarity of human brain morphology. Our results showed that emotional well-being was significantly correlated with global centrality measurements: negatively correlated with the betweenness centrality in the right visual network but positively correlated with the eigenvector centrality in the right precentral sulcus; total score of well-being was positively correlated with the local efficiency in the right posterior cingulate cortex and the right precuneus of cortical thickness network. We inferred that emotional well-being was involved in more preliminary processing stages including perceptual and attentional information, and hedonic and eudaimonic well-being might share some common morphological network mechanisms in the subsequent advanced cognitive processing stages.

### The role of the visual network and the precentral sulcus in emotional well-being

Our results showed that emotional well-being was significantly correlated with global centrality measurements: negatively correlated with the betweenness centrality in the visual network but positively correlated with the eigenvector centrality in the precentral sulcus of cortical thickness network. Emotional well-being, also known as subjective or hedonic well-being, is comprised of two components: an affective component, which refers to positive effects and affect balance; and a cognitive component, which refers to participants’ levels of life satisfaction and cognitive control [7, 9, 55]. Considering the affective component, people with high well-being tended to pay more attention to positive emotional expression [56] while people with low well-being were sensitive to unpleasant feedback and showed impaired attention to adverse results [57]. Considering the cognitive component, researchers proposed people with high or low levels of well-being would apply various cognitive and motivational processing strategies such as perceptional and emotional processing [58]. For instance, people with high well-being had a strong ability of emotional regulation and resilience [56], while people with low well-being were sensitive to negative implications and showed self-focused cognition (rumination) [57].

Accordingly, on one hand, the visual network mainly spans across the visual cortex including the occipital and the lingual gyrus as well as the cuneus. Previous studies demonstrated the cuneus was functionally connected to the visual network and played a role in visual information integration [59], and the lingual gyrus was associated with the fear network [60, 61] and identification of facial emotional expressions [62, 63]. The visual cortex including the occipital cortex was involved in conscious processing [64], processing of uncertain cues [65] as well as perceptual processing [66]. Killgore and Yurgelun-Todd also demonstrated the activations of parts of the visual network were correlated with stress from social interactions [67]. Thus, the negative correlation between betweenness centrality in the visual network and emotional well-being indicated that compared with happy individuals, individuals with low emotional well-being might have a tendency to show more perception and conscious attention to uncertain cues or fear events arousing negative emotions and feelings.

On the other hand, the precentral sulcus is typically segmented into two parts including the superior and the inferior precentral sulcus and these two parts develop independently [68]. Previous studies referred to both regions as the frontal eyes field (FEF) because both of them were activated by saccade tasks [69–71]. The inferior precentral sulcus was mainly involved in auditory and visual attention as well as short-time memory [72–74]. And the FEF in the precentral sulcus was not only involved in preparing and triggering various eye movements [75–78] but also in various preliminary cognitive processing including attention orientating and visual awareness [79–82]. Therefore, the positive correlation between emotional well-being and the eigenvector centrality in the inferior precentral sulcus indicated the central role of the precentral sulcus in enhancing participants’ emotional well-being, and participants with higher emotional well-being might be inclined to pay more perceptual (auditory and visual) attention to dwell about positive implications of events and circumstance to enhance their levels of well-being.

In a summary, our results demonstrated compared to unhappy people, people with high emotional well-being would apply various preliminary cognitive processing strategies such as perception (visual and auditory) processing and attention orientating to more positive feedback and aspects of events, to maintain and enhance their levels of emotional well-being, which was in line with the construct theory of well-being [58].

### The role of the precuneus and the posterior cingulate cortex in total score of well-being

We also found total score of well-being was positively correlated with the local efficiency in the RH_Default_PCC [the precuneus and the posterior cingulate cortex (PCC)] of cortical thickness network, which was consistent with previous studies [24, 32, 33]. The local efficiency was at the spatial scale of local, compared with the above global measurements associated with emotional well-being. The PCC and the precuneus were parts of the default mode network [83], and they were related to multiple cognitive processing including identification of self and emotional states of others [84], construction of past, present, and future selves [36], autobiographical and retrospective memory [36] as well as self-reflection [85]. In more detail, on the one hand, the PCC was involved in a series of memory-based construction/simulation functions including autobiographical memory, situational future thinking, and scene construction [83]. Previous studies also demonstrated the correlations between impairments of the PCC and the degeneration of scene construction capacity in Alzheimer’s disease [86]. Meanwhile, the PCC was also involved in a series of cognitive processes including emotional processes [87], memory retrieval [88], self-referential [89–91] and self-reflection [85]. On the other hand, the precuneus, as a part of the medial posterior parietal cortex, was also involved in an array of cognitive processing including self-related processing [92, 93], conscious information processing [94], episodic memory [95] and visuospatial processing [96]. Previous studies also demonstrated the anatomical and functional abnormalities of the precuneus in various diseases including Alzheimer’s disease [97–99], Huntington’s disease [100], and mild cognitive impairment [101–103].

Total score of well-being can be subdivided into two dimensions of well-being, hedonic well-being, also known as emotional well-being, and eudaimonic well-being (including psychological and social well-being) [10]. These two dimensions are positively correlated but distinct components [9, 11, 104]. Previous studies have proved that compared to hedonic well-being, people with high eudaimonic well-being tended to spend more time on self-reflection to identify selves’ true value and integrate past, present, as well as future events to maintain and realize their levels of well-being [104, 105]. While people with high hedonic well-being were inclined to focus on positive emotional events [56] and showed less attention to adverse results [57] and would apply multiple cognitive and motivational processing strategies such as self-reflection, perception processing, and emotional processing to maintain their levels of well-being [58]. These characteristics of hedonic and eudaimonic well-being were in line with the functions of the precuneus and the PCC in the default mode network mentioned above. We inferred that people with either hedonic or eudaimonic well-being would apply some similar cognitive strategies in subsequent advanced processing procedures possibly for different goals. Thus, the cognitive processes of these two regions reflected both dimensions of well-being in high levels of autonomy, self-acceptance, self-reflection, various memory, scene construction as well as emotional processing in pursuits of positive affect (hedonic well-being) and actualization of one’s potential or true value (eudaimonic well-being) [7, 8].

In conclusion, in the study, we applied a novel method to construct human brain morphological similarity network to explore topological mechanisms of well-being. We confirmed that different dimensions of well-being were associated with human brain morphological similarity network topology at different spatial scales. Our results provided compelling evidence of the ability of information transfer and the central role of the visual network, the precentral sulcus, the precuneus and the posterior cingulate cortex in different dimensions of well-being, which were involved in various cognitive processes. We inferred that emotional well-being was involved in more preliminary processing stages including perceptual and attentional information processing, as well as hedonic and eudaimonic well-being might share some common morphological network mechanisms in the subsequent advanced cognitive processing stages.

### Limitations

Several limitations should be taken into consideration: (1) The sample size was small but across a big age span, and the generalizability of its results should be tested in future big sample; (2) Also, well-being was a multifaceted and complex component, and was also related to various brain regions involving subcortical structures. The present study only explored human brain information of cerebral cortex but did not involve the exploration and analysis of subcortical structures; (3) The behavioral data were collected from participants’ self-reports, which were largely influenced by participants’ subjective feelings and emotions. More objective measurements should be applied in future studies.

## Methods

### Participants

Participants were recruited from local community by advertisements. The initial sample included 67 datasets (32 males and 35 females; mean age = 32.79 ± 13.11; ranged from 18.59 to 64.30). All the participants were invited for a detailed mental health interview using the Mini-International Neuro-Psychiatric Interview and people with a history of or current major neuropsychiatric illness, head injury, alcohol, drug abuse were excluded from the study. We also excluded people with MRI contraindications, including people with implants, pacemakers, brain surgery, current pregnancy, and very recent tattoos. In addition to the MRI scanning, the participants were also assessed with the Short Form of the Mental Health Continuum (Chinese). The final sample included 65 datasets. Participants who were absent from the MRI scanning (n = 1) or didn’t pass the mental health interview (n = 1) were excluded. The institutional review board of the Institute of Psychology Chinese Academy of Sciences approved this study and written informed consent was obtained from participants before data acquisition.

### Behavior measurements

The Short Form of the Mental Health Continuum in Chinese (MHC-SF) was applied to measure participants’ well-being. The 14-item version was based on a model comprising three components of well-being [8, 106]: emotional well-being (3 items including positive affects and avowed quality of life), psychological well-being (6 items including self-acceptance, personal growth, purpose in life, positive relations with others, autonomy, and environmental mastery) and social well-being (5 items including social contribution, social integration, social actualization, social acceptance, and social coherence). It includes items like, “I feel happy” (Emotional well-being), “I like most parts of my personality” (Psychological well-being), “I feel close to other people in my community” (Social well-being). Participants were asked to respond on which ‘1’ represented ‘never’ and ‘6’ represented ‘every day’ in the item according to the frequency of experiencing various symptoms of well-being in the past month. The scores of each dimension were calculated by summing the scores of the items belonging to them. Higher scores indicate higher levels of well-being. The Chinese version has high reliability and validity, via a 6-point scale for all items and describes the frequency of experiencing various symptoms of well-being [107]. The Cronbach’s α coefficient in the present study was 0.944.

### Imaging acquisition

All the MRI images were collected on a GE 3.0 T scanner (Discovery MR750) at the Institute of Psychology Chinese Academy of Sciences. Participants completed a T1-weighted structural MRI scan (eyes closed) with a magnetization-prepared rapid gradient-echo (MPRAGE) sequence with the following parameters: repetition time (TR) = 6.652ms, echo time (TE) = 2.928ms, inversion time (T1) = 450ms, flip angle (FA) = 12°, field of view = 256 × 256 mm^2^ and acquisition matrix = 256 × 256, slice thickness = 1.0mm, 192 sagittal slices, voxel size = 1 × 1 × 1 mm^3^.

### Imaging data preprocessing

MRI images were preprocessed by the Connectome Computation System (CCS) (http://github.com/zuoxinian/CCS), which was developed by our laboratory [108] integrating several software including AFNI [109] (Analysis of Functional NeuroImages), FSL [110] (fMRI Software Library), and FreeSurfer [111]. The CCS pipelines were employed to preprocess all individual structural images as well as quality control [108, 112]. The structural images preprocessing included (1) noise removal and brain extraction (skull stripping) using volBrain automated volumetry system (http://volbrain.upv.es) [113]; (2) image intensity inhomogeneity correction; (3) tissue segmentation of cerebrospinal fluid (CSF), white matter (WM) and deep gray matter (GM); (4) generation of the GM-WM (white surface) and GM-CSF interface (pial surface); (5) spatial registration via matching of the cortical folding patterns across participants by recon-all in FreeSurfer and Gaussian spatial smoothing (FWHM = 6mm, Full Width at Half Maxima); (6) Finally, the 3D (dimensional) structure images were projected onto the fsaverage5 standard cortical surface with 10,242 vertices per hemisphere.

### Quality control procedure

Quality control is very significant for solid data analysis. The CCS provided quality control procedures for both functional and structural images. For structural MRI in this study, the quality control procedure (QCP) was as follows: (1) brain extraction or skull stripping; (2) image tissue segmentation; (3) reconstruction of pial and white surface; and (4) head motion. We performed the visual inspection on all the original structural images and excluded participants with obvious structural brain abnormalities and significant motor artifacts during the scan. The CCS provides screenshots of the brain tissue segmentation as well as screenshots of pial and white surface reconstruction. We visually checked the screenshots, and participants with bad brain tissue segmentation and surface reconstruction were excluded from the subsequent analysis. All the participants passed the quality control. The final sample included 65 participants and their descriptive information and inter-variable correlations were shown in Table 1.

### Morphological similarity network construction

In the study, we used a macro-scale brain network parcellation, which subdivided the entire cortical surface into 51 spatially connected parcels which were derived from a clustering approach on MRI images of 1000 subjects to identify networks of functionally coupled regions across the cerebral cortex [114], to construct human brain morphological network based on their distributions, and then we calculated mean cortical thickness of each parcel. We excluded the parcels whose vertex number was less than 50, and finally got 32 parcels reserved for final group analysis: expanding across all the Yeo-7 networks: visual network, somatomotor network, dorsal attention network, ventral attention network, limbic network, frontoparietal (control) network, and default mode network (see Table 2).

**Table 2:** The vertex number of reserved 32 brain regions

Brain region	Vertex number	Brain region	Vertex number
LH_Vis	1213	RH_Vis	1264
LH_SomMot	1590	RH_SomMot	1612
LH_DorsAttn_Post	627	RH_DorsAttn_Post	614
LH_DorsAttn_FEF	97	RH_DorsAttn_FEF	98
LH_SalVentAttn_ParOper	130	RH_DorsAttn_PrCv	50
LH_SalVentAttn_FrOper	331	RH_SalVentAttn_TempOccPar	208
LH_SalVentAttn_Med	216	RH_SalVentAttn_FrOper	313
LH_Limbic_OFC	213	RH_SalVentAttn_Med	242
LH_Limbic_TempPole	328	RH_Limbic_OFC	237
LH_Cont_Par	151	RH_Limbic_TempPole	318
LH_Cont_PFCl	291	RH_Cont_Par	167
LH_Default_Par	263	RH_Cont_PFCl	543
LH_Default_Temp	359	RH_Default_Par	183
LH_Default_PFC	771	RH_Default_Temp	269
LH_Default_PCC	275	RH_Default_PFCv	60
		RH_Default_PFCm	461
		RH_Default_PCC	225

As in our previous study [47], we estimated distribution similarity of cortical thickness for each pair of parcels to construct human brain morphological similarity network. Firstly, for each pair of parcels, we segmented both of their cortical thickness into 30 bins. Secondly, we calculated the vertex frequency for each bin of the two parcels, and then we got the frequency distribution histogram for each parcel. Finally, we computed the Pearson’s correlation to estimate the similarity of cortical thickness distribution, and then we obtained a 32 × 32 morphological correlation matrix for each participant. There were both positive and negative connections between different brain regions which respectively demonstrated co-varying and anti-correlated distribution curves, and the negative connections only occupied a tiny proportion of the entire connection matrix. Therefore, in the study, we considered the absolute values of connections to computing network topological measurements considering the little effects of negative connections on the whole brain network topology. Then, we used orthogonal minimal spanning trees (OMST) analysis, which was a threshold-free method to derive the strongest connections of a network and reserve important information about brain network organization [115], to get an undirected weighted graph, and then the topological measurements could be computed based on the binary (unweighted) correlation matrix.

### Topological measurements

We computed network efficiency including global efficiency (Eglob), nodal efficiency (Enodal) and local efficiency (Elocal) as well as network centrality including degree centrality (DC), betweenness centrality (BC), eigenvector centrality (EC) and pagerank centrality (PC) based on the binary (unweighted) correlation matrix using the Brain Connectivity Toolbox (http://www.brain-connectivity-toolbox.net) [48] and the CCS scripts [108].

#### Network efficiency

Global efficiency for network *G* is defined as:

1 $${E}_{glob}\left(G\right)=\frac{1}{N(N-1)}\sum_{i,j,i\ne j\in G}\frac{1}{{L}_{ij}}$$

where *N* is the number of nodes and *L*_*ij*_ is the shortest path length between node *i* and node *j* in graph *G* [52]. Global efficiency is a global measurement of the parallel ability of information transfer within the whole network.

Nodal efficiency of node* i* is defined as:

2 $${E}_{nodal}(i)=\frac{1}{N-1}\sum_{j,i\ne j\in G}\frac{1}{{L}_{ij}}$$

where *N* and *L*_*ij*_ are the same as that in Eq. (1), respectively representing the number of nodes and the shortest path length between node *i* and node *j* in graph *G*. Nodal efficiency measures the ability of the node for information transfer within the whole network and is also a global measurement.

Local efficiency of node *i* is defined as:

3 $${E}_{local}(i)={E}_{glob}\left({G}_{i}\right)$$

where *G*_*i*_ is a subgraph and is composed of the nodes that connect to node *i* (not including node *i*) directly and interconnected edges. Local efficiency indicates how well the information is exchanged in the given brain region and hence is a local measurement.

#### Network centrality

Degree centrality of node *i* is defined as:

4 $$DC\left(i\right)=\sum_{j\in N}{a}_{ij}$$

where *N* is the set of all nodes in the network, and *a*_*ij*_ is the connection status between* i* and *j*: *a*_*ij*_ = 1 when i and j were connected and *a*_*ij*_ = 0 when i and j weren’t connected. DC identifies the nodes with the most connected links and is the most common quantifiable local centrality measure [48, 49, 116].

Betweenness centrality of node *i* is defined as:

5 $$BC\left(i\right)=\sum_{k,j\in N, k\ne j,k\ne i,i\ne j}\frac{{L}_{kj}(i)}{{L}_{kj}}$$

where *L*_*kj*_ is the number of shortest paths between node *k* and node *j*, and *L*_*kj*_(*i*) is the number of shortest paths between *k* and* j* that pass through node *i*. BC represents the fraction of all shortest paths in the network that pass through a given node. High BC indicated the nodes were important in connecting disparate parts of the network [48, 117] and were global measuremens.

Eigenvector centrality of node *i* is defined as:

6 $$EC\left( i \right) = \mu_1 \left( i \right) = \frac{1}{\lambda_1 }\mathop \sum \limits_{j = 1}^N a_{ij} \mu_1 (j)$$

where ${\mu }_{j}\left(i\right)$ is the *i*-th component of the *j*-th eigenvector of the adjacency matrix* a*_*ij*_, and ${\lambda }_{1}$ is the corresponding *j*-th eigenvalue.* N* is the set of all nodes in the network, and *a*_*ij*_ is the connection matrix. EC considers the nodes connecting to other high degree nodes as highly central and indicates a central and important role of the node in the network [118, 119].

Pagerank centrality of node* i* is defined as:

7 $$PC\left( i \right) = r\left( i \right) = 1 - d + d\mathop \sum \limits_{j = 1}^N \frac{{a_{ij} r(j)}}{DC(j)}.$$

Pagerank centrality was introduced originally by Google to rank web pages. In graph theory, PC represents the importance of nodes assuming that the importance of a node is the expected sum of the importance of all connected nodes and the direction of edges [120, 121]. The PC algorithm is a variant of EC, which introduces a small probability (1—d = 0.15, d is damping factor) of random damping to handle walking traps on a graph [122]. Both EC and PC are global centrality measurements.

### Statistics

To investigate the associations between topological measurements (i.e., network efficiency Effi and centrality Cent) of human brain morphological similarity network and different dimensions of well-being, we applied general linear model that took age, sex, education, intracranial volume (ICV), mean cortical thickness (CT) as covariates. The detailed statistical model was shown in Eq. (8).

8 $$Well-being\,=\, {\alpha }_{1}\times age+{\alpha }_{2}\times sex+{\alpha }_{3}\times education+{\alpha }_{4}\times ICV+{\alpha }_{5}\times {CT}_{mean}+\beta \times Effi/Cent+\gamma$$

False discovery rate (FDR, q < 0.05) correction for 32 parcels was used to control type 1 error over multiple tests. And the General Linear Model statistical analysis and FDR correction were performed using MATLAB scripts in the study.

## Data Availability

https://github.com/stronger202203/HumanBrainNetworkTopology.
